# Cues to intention bias action perception toward the most efficient trajectory

**DOI:** 10.1038/s41598-019-42204-y

**Published:** 2019-04-18

**Authors:** Katrina L. McDonough, Matthew Hudson, Patric Bach

**Affiliations:** 1 University of Plymouth, School of Psychology, Plymouth, PL48AA UK; 2 School of Business, National College of Ireland, Mayor Street, Dublin 1, Ireland

**Keywords:** Perception, Human behaviour

## Abstract

Humans interpret others’ behaviour as intentional and expect them to take the most energy-efficient path to achieve their goals. Recent studies show that these expectations of efficient action take the form of a prediction of an ideal “reference” trajectory, against which observed actions are evaluated, distorting their perceptual representation towards this expected path. Here we tested whether these predictions depend upon the implied intentionality of the stimulus. Participants saw videos of an actor reaching either efficiently (straight towards an object or arched over an obstacle) or inefficiently (straight towards obstacle or arched over empty space). The hand disappeared mid-trajectory and participants reported the last seen position on a touch-screen. As in prior research, judgments of inefficient actions were biased toward efficiency expectations (straight trajectories upwards to avoid obstacles, arched trajectories downward towards goals). In two further experimental groups, intentionality cues were removed by replacing the hand with a non-agentive ball (group 2), and by removing the action’s biological motion profile (group 3). Removing these cues substantially reduced perceptual biases. Our results therefore confirm that the perception of others’ actions is guided by expectations of efficient actions, which are triggered by the perception of semantic and motion cues to intentionality.

## Introduction

Humans see others’ behaviour as purposeful and goal directed^1–4^. A key signature of this “intentional stance”^5^ is the assumption that other people generally act rationally: they take the most energy-efficient path to achieve their goal, and expend additional energy only when an obstacle has to be overcome^3,6^. This simple heuristic of action efficiency arises early in development and allows children to attribute intentionality to observed behaviours, even when carried out by inanimate objects^7–9^. Human infants (and some non-human primates) show surprise, for example, when actors that are believed to be intentional violate these assumptions, such as when they do not try to avoid an obstacle or exert additional unnecessary energy to reach their goal^4,10^. Once established, this simple heuristic may form a stepping-stone for more sophisticated abilities for reasoning about others^4,11^. For example, observing an inefficient action (e.g. reaching directly towards an object despite an obstacle in the way) can help people realize that others act according to beliefs and not objective reality (i.e. they may not have sight of the obstacle), forming the basis of a prototypical theory of mind.

We have argued that these expectations of efficient action are, to some extent, perceptually represented, in the form of an ideal “reference” trajectory that a rational actor would take through a given environment, against which observed actions can be judged^12,13^. This proposal emerges from recent predictive processing accounts of social perception^12,14–17^ which argue that perception of others’ actions – like perception in general – is always hypothesis-driven. Any assumption about the external world (and the people within it) is translated into the perceptual input that would result from such a state. These expectations of future input can guide perception and be tested against actual stimulation^18–20^. In non-social perception, such expectations explain several visual illusions (e.g., dress illusion^21^), the switch between different bi-stable percepts^22^, or why the same objects can appear convex or concave depending on prior assumptions about light sources^23^. In social perception, simply attributing a goal to another person could similarly elicit predictions about how this individual would realise such a goal, specifying which action they may soon carry out^14–16,24,25^ (for theoretical arguments, see^26,27^). The principle of efficient action can make a direct contribution here, specifying the ideal “reference” trajectory that achieves the actor’s goals with minimum energy expenditure, given the current environmental constraints, such as potential obstacles in the way^12,26^.

In a recent series of studies, we attempted to reveal these expectations of efficient action^13^. These studies relied on the well-established phenomenon that the uncertainty during motion perception is perceptually sharpened using top-down information^28,29^, filling in missing information^30–32^ in a predictive manner^33,34^. The resulting perceptual biases can be reliably measured by suddenly removing the moving object from view, and asking participants to report its disappearance point, either on a touch screen^35–37^ or by comparing it to probe stimuli presented shortly after^38–42^. In such a paradigm, people generally over-estimate the movement they have seen, reporting the moving stimulus to have disappeared further along its trajectory than it really did (i.e. the representational momentum effect^38,39^). These displacements have been shown to not only reflect a simple extrapolation of motion based on the previously seen trajectory^43^, but also prior knowledge about it’s causes, such as how the motion would be affected by one’s own actions^44^, by physical forces such as friction or gravity^45^, or the most likely behaviours of the other person^41,42,46–48^.

In the case of observed actions, the perceptual biases reflect the predictions derived from the assumption of efficient action^13^. In a recent series of experiments, participants observed a hand starting to reach for an object with a straight or arched trajectory. The actions were either efficient (reaching straight when the path was clear or arched over an obstacle) or inefficient (straight towards an obstacle or arched over empty space). The movement disappeared before it was completed and participants reported the hand’s last seen position on a touch screen, or by comparing it to probe stimuli presented immediately after. Both measures revealed that perceptual judgements were reliably biased by expectations of efficient action. Straight reaches were reported to have reached higher when an obstacle was blocking its path, in line with the expectation that the hand would soon lift to avoid it. Conversely, high arched reaches were reported lower when no obstacle was present, and corrected towards the straighter, more energy-efficient trajectory. These biases were present automatically, but increased when participants explicitly predicted–prior to action onset–the most efficient trajectory through the scene, or when attention was drawn to the environmental constraints. Moreover, they could be disrupted by dynamic visual noise masks presented directly after stimulus offset, suggesting that the biases emerge during ongoing perception or directly after the sudden offset, when the visual system “fills in” the expected future path^49,50^.

Together, these results indicate that the teleological stance is at least partly perceptually represented, providing an ideal reference trajectory that informs the action that was indeed perceived. Here, we test on what stimulus features these predictions of efficient action depend. In children, as well as in adults, intention attribution – and the resulting surprise when seeing an inefficient action–depends on the presence of cues to intention^51–53^, such as seeing an agentive stimulus (such as a hand relative to a ball^54^), or observing movements with biological motion trajectories^55–59^. If such cues indeed trigger attributions of intentionality to others, and the expectation of efficient action is tied to such intentionality attribution, then they should also determine to what extent perceptual biases towards efficient actions are observed.

In the first experimental group, we replicated the original experiment by Hudson and colleagues^13^, in which participants saw efficient and inefficient reach trajectories (arched/straight over an obstacle vs. empty space) and indicated the hand’s last location after it had suddenly disappeared on a touch screen. In two further experimental groups, we progressively removed intentional cues. First, as in prior research on infant intention attribution^54^, we replaced the hand with a non-agentive stimulus – a ball –, which however followed the same characteristic biological motion trajectories and profiles as the hands in the first experimental group, showing the classical bell-shaped velocity profile of reaches towards objects^60^. Second, humans are sensitive to motion cues that distinguish the intentional biological agents from inanimate objects, such as self-propulsion and change of direction^55,58^, or a trajectory and speed of movement that is similar to one’s own movement^56,57,59^. In a third group, participants therefore saw the same ball, but it did not now follow a biological motion profile, removing all kinematic cues to intention. If biases toward efficient action emerge from cues that signal intentionality, then they should be substantially reduced in group 2, and further reduced in group 3, as cues to intentionality are removed.

## Method

### Participants

Eighty-two participants took part in the experiment: twenty-nine participants in group 1 (hand stimuli, mean age = 21 years, SD = 4.7, 25 females), twenty-seven in group 2 (balls with biological motion, mean age = 20 years, SD = 4.1, 21 females), and twenty-six in group 3 (balls with non-biological motion, mean age = 21 years, SD = 4.2, 20 females). Nine additional participants (two from group 1, three from group 2, four from group 3) were excluded based on previously established exclusion criteria (see Results). All participants in all groups were tested in the same three-week period. Both the gender mix and age distribution did not differ between groups (*p*s > 0.39). All participants were right-handed, had normal/corrected-to-normal vision, gave informed consent, and were recruited from the University of Plymouth or the wider community for course credit or payment. The study was approved by the University of Plymouth’s ethics board, in line with the ESRC and the Declaration of Helsinki. A power analysis revealed that a sample size of 26 provides 0.80 power to detect two-sided within-subjects effects in each of the group with Cohen’s *d* = 0.57. Our prior study investigating the same effect^13^ (“report obstacle” condition) and pilot data revealed consistently larger effect sizes, *d* = 0.76 to *d* = 1.29. For the between-subjects effects, a power analysis revealed that a sample size of 26 per group provides 0.80 power to detect effects in either direction with Cohen’s *d* = 0.79. This should provide enough power to detect reductions of the original effect to about 40% of the original size (assuming that standard deviations remain the same).

### Apparatus

Presentation (NeuroBS) software was used to present the experiment via a HP EliteDisplay S230tm 23-inch widescreen (1920 × 1080) Touch Monitor. Verbal responses were recorded with Presentation’s sound threshold logic via a Logitech PC120 combined microphone and headphone set.

### Stimuli

**Figure 1:**
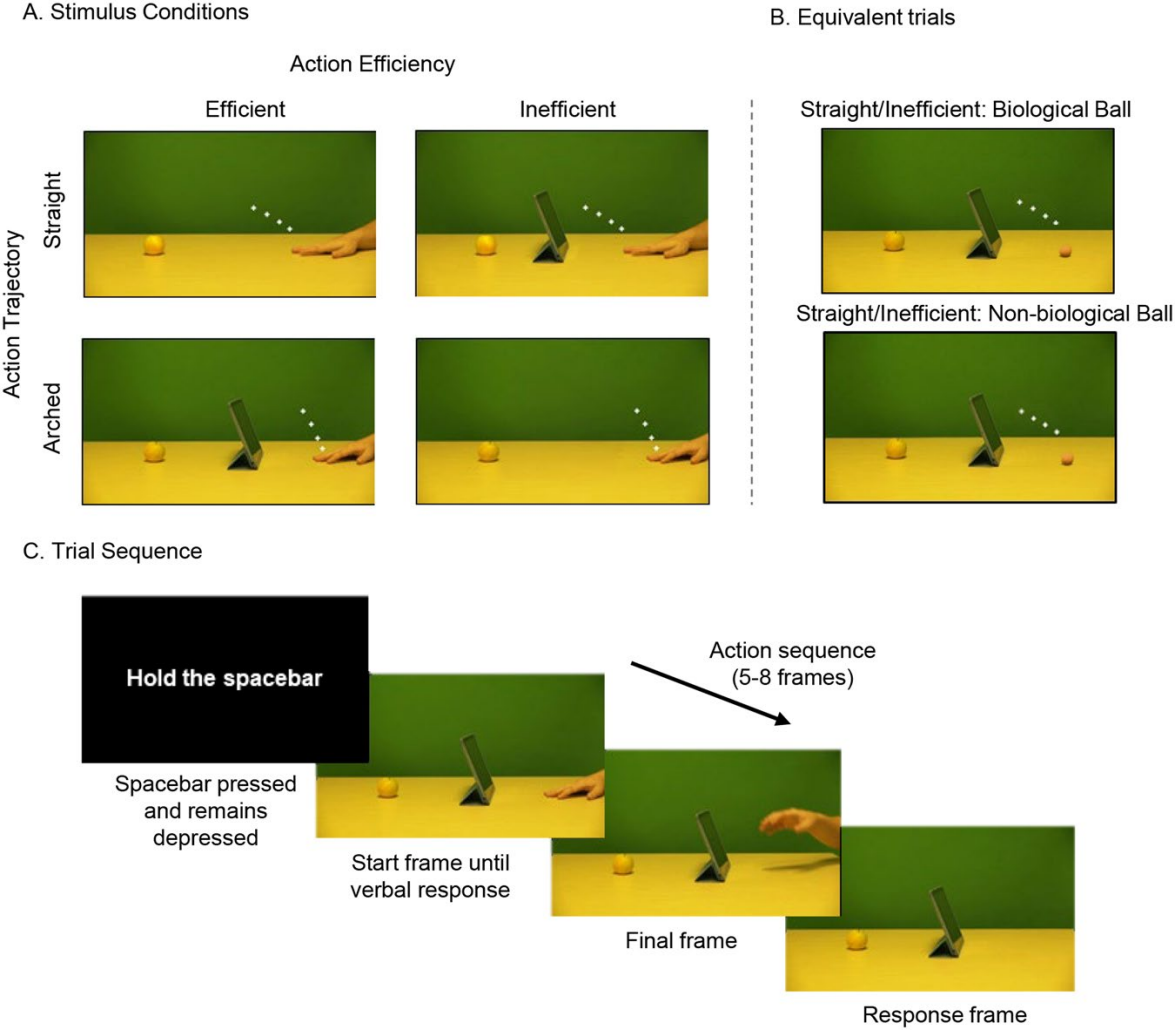
Stimulus conditions and trial sequence. The stimulus conditions used in all three experimental groups are depicted in Panel A. The four panels show the hand in the starting position and the possible action trajectories. These Action Trajectories  were either straight or arched and were rendered either efficient or inefficient by the presence or absence of an obstructing object. Panel B depicts an equivalent example of a Straight/Inefficient trial in the Biological Ball group (top) and the Non-Biological Ball group (bottom). The white markers depict the disappearance point of the index finger tip/ball in each of the four final frames. Panel C shows an example of a trial sequence in the Arched/Efficient condition of group 1. This trial sequence is equivalent across all experimental groups.

Example stimuli can be seen in Fig. 1A. To derive a set of stimuli of efficient actions, videos were filmed of an arm at rest to the right of the screen, which then reached for one of four objects (an apple, a packet of crisps, a glue stick, or a stapler) on the left of the screen. The reaches were either directed straight for the target object (Straight/Efficient), or arched over one of three obstacles (an iPad, lamp or pencil holder; Arched/Efficient). Each video clip was then converted into individual frames, and the first 22 from frame 1 (initial rest position) to 22 (mid-way through the action) were used as stimuli. For each efficient action, an inefficient action sequence was created by digitally removing the obstacles from the Arched/Efficient videos (Arched/Inefficient), or by inserting the obstructing objects into the Straight/Efficient videos, (Straight/Inefficient). The inefficient actions were therefore identical to the efficient actions in terms of movement kinematics, and differed only by the presence/absence of the obstacle. Finally, response stimuli were created by taking one frame from each action sequence and digitally removing the actor’s arm from the scene, so that only the objects and background remained. Presenting this frame immediately after the action sequence gave the impression of the hand disappearing from the scene, and participants indicated the last seen location of the tip of the index finger on this frame with a touch response on screen.

For experimental group 2 (balls with biological motion), the forty videos of hand movements used in experimental group 1 were digitally manipulated so that the actor’s hand was replaced with a ball, coloured using the same tones as the hand. The ball was the same size as the tip of the index finger that participants had to touch in experimental group 1 (30 px. diameter) and was positioned at the same coordinates in each frame. An additional frame was created by positioning the ball mid-air before the first frame (where the ball contacts the table) creating an illusory “bounce” motion, providing a realistic context for the ball movement in order to reduce impressions of self-propelled movement that could also cue the observer that the motion is intentional^61^.

For Experimental group 3 (balls with non-biological motion), the forty videos from group 2 were digitally manipulated so that the ball now appeared to move in a straight line and at a constant speed after the bounce frame, eliminating the biological motion profile. To ensure that comparability of disappearance points between experimental groups, the line of best fit was calculated through the last four frames of each sequence of experimental group 1 (i.e. all possible disappearance points). The constant speed of the ball was created by recalculating the Y coordinates at equal distances along this line, between the first and last frame.

### Procedure

An example trial sequence can be seen in Fig. 1B. Participants completed four blocks of 48 trials in which each condition was presented an equal amount of times (Straight/Efficient, Straight/Inefficient, Arched/Efficient, Arched/Inefficient). At the start of each trial, participants saw an instruction to “Hold the spacebar”, to which they pressed the spacebar with their right hand and kept it depressed. This ensured that they did not track the observed motion with their finger and could only initiate their response once the action sequence had disappeared. Participants then saw the first frame of the action sequence as a static image (the hand at rest in experimental group 1 and the “bouncing ball” frame in experimental groups 2 and 3) and were required to say “yes” into the microphone if there was an obstructing object present, and “no” if there was not.

The action sequence began 1000 ms after a verbal response had been detected. Every third frame of the action sequence was presented for 80 ms each, with a randomly selected sequence length of 5, 6, 7 or 8 frames (e.g. trials with a length of 8 frames showed frames 1-4-7-10-13-16-19-22). The final frame was then immediately replaced with the response frame, which showed the same scene without the moving object, creating the impression that the hand/ball had simply disappeared. Participants released the spacebar and, with their right hand, touched the screen where they thought the final position of the tip of the observed index finger was in group 1, or the final ball position in groups 2 and 3. As soon as a response was registered, the next trial began.

Note that the presentation of every third frame of the videos resulted in illusory “apparent” motion between the steps in the trajectory^62^. Such non-smooth motion retains the relevant characteristics of intentional biological motion (e.g. parabolic path, bell-shaped velocity profile) and provides ideal conditions to measure predictive influences in motion perception, which are larger with apparent motion than smooth motion^63^. This is in line with the notion that top-down influences that govern everyday perception become apparent the more the bottom-up sensory input becomes ambiguous or uncertain (e.g. through bi-stable images^64^; visual noise^65^). For motion, non-smooth step-wise presentation is assumed to disrupt low-level motion detectors, prompting a stronger weighting of top-down influences that compensate and “fill in” the intervening steps in the trajectory^30,32,66^.

## Results

Data filtering was identical to our original study^13^. In all three experimental groups, trials were excluded if the correct response procedure was not followed (e.g. lifting the spacebar too early; 3.5%), or if response initiation or execution times were shorter than 200 ms or more than 3 SDs above the sample mean (2.2%, Initiation: mean = 393.7 ms, *SD* = 173.3; Execution: mean = 571.9 ms, *SD* = 203.3). Participants were excluded if too few trials remained after trial exclusions (<50% valid trials, 3 participants), if the distance between the real and selected positions exceeded 3 SD of the sample mean (mean = 39.9 pixels, *SD* = 18.9, 2 participants excluded), or if the correlation between the real and selected positions was more than 3 SD below the median r value (X axis: median *r* = 0.940, *SD* = 0.041; Y axis: median *r* = 0.908, *SD* = 0.063, 4 participants excluded).

Analysis was conducted on the predictive perceptual bias by subtracting the real final coordinates of the tip of the index finger/ball from the participant’s selected coordinates on each trial. This resulted in separate “difference” scores along the X and Y axis where positive X and Y scores represented a rightward and upward displacement respectively, and negative X and Y scores represented a leftward and downward displacement respectively. A score of 0 on both axes indicated that the participant selected the real final position exactly. These difference scores were entered into a 2 × 2 × 3 ANOVA for the X and Y axis separately, with Trajectory (straight vs arched) and Efficiency (efficient vs inefficient) as repeated-measures factors, and Experimental group as a between-subjects factor.

The data from the original experiments^13^, as well as further pilot studies in our lab, have shown that expectations of efficient action primarily induce biases on the Y-axis, but not the X-axis. This is consistent with the view that rather than viewing the current trajectory relative to the trajectory that was initially predicted (e.g. an arched trajectory when an obstacle was present), expectations of action efficiency reflect expectations about how the current trajectory will further develop. In other words, when seeing a straight reach towards an obstacle, one expects the hand to be merely lifted upwards to avoid the obstacle (rather than it being also displaced backwards to its corresponding location had it followed the arched trajectory from the outset). Similarly, when seeing an arched reach over empty space one expects the current reach would straighten downwards towards the goal object (rather than also being displaced forwards to where the hand would be had it followed the alternative straight trajectory). If the current results replicate this established pattern^13^, displacement should therefore again primarily affect the Y axis (capturing this lifting or lowering of the hand towards the target or away from the obstacle), but not the X-axis (indexing a displacement forwards/backwards to the alternative trajectory).

### Y axis

If intentionality is perceptually instantiated, we predicted (1) that inefficient actions would be perceptually “corrected” towards the more efficient action alternative, and (2) that these biases should be strongest in experimental group 1 (hand stimuli) but weaker when cues to intentionality are removed in groups 2 (balls with biological motion) and 3 (balls with non-biological motion). Indeed, the analysis revealed the interaction of Trajectory and Efficiency (*F*(1,79) = 45.0, *p* < 0.001, *η*_*p*_^2^ = 0.363), replicating our prior study^13^. Across groups, the disappearance points for straight trajectories were reported higher when the actions were inefficient (i.e. reaching towards an obstacle, 2.26 px) than when the actions were efficient (no obstacle, −0.967 px; *t*(81) = 5.46, *p* < 0.001, *d* = 0.60). Conversely, the perceived disappearance points for arched reaches were perceived to be lower for inefficient actions (7.87 px) than for efficient actions (11.6 px; *t*(81) = 4.81, *p* < 0.001, *d* = 0.53).

Importantly, as predicted, these biases differed between experimental groups, as indicated by an interaction of Trajectory, Efficiency and Experimental group (*F*(1,79) = 6.47, *p* = 0.002, *η*_*p*_^2^ = 0.141). Pairwise step-down comparisons showed that the interaction between Trajectory and Efficiency was smaller in the Non-biological Ball group (group 3) than in the Biological Hand group (group 1; *F*(1,53) = 11.7, *p* = 0.001, *η*_*p*_^2^ = 0.181), and the Biological Ball group (group 2; *F*(1,51) = 4.00, *p* = 0.051, *η*_*p*_^2^ = 0.073). No difference was found between Biological Hand group and the Biological Ball group (*F*(1,54) = 2.77, *p* = 0.102, *η*_*p*_^2^ = 0.049), although a Two One-Sided Tests (TOST) procedure^67^ indicated that the observed effect size (*d* = 0.45) was not significantly within the equivalence bounds of ΔL = −0.53 and ΔU = 0.53, *t*(53.85) = −0.31, *p* = 0.38 (equivalence bounds calculated as critical Cohen’s d-values from our prior study^13^ investigating the same effect^67^). When experimental groups were analysed separately, the interaction between Trajectory and Efficiency was only present for the groups seeing Hands and Balls on biological motion trajectories (Biological Hand: *F*(1,28) = 41.7, *p* < 0.001, *η*_*p*_^2^ = 0.598; Biological Ball: *F*(1,26) = 21.0, *p* < 0.001, *η*_*p*_^2^ = 0.447), but not in the group viewing balls on a non-biological motion trajectory (Non-biological Ball: *F*(1,25) = 1.20, *p* = 0.284, *η*_*p*_^2^ = 0.046). Indeed, the TOST procedure indicated that the observed effect size in the latter group (*d* = 0.21) was significantly within the equivalence bounds of ΔL = −0.55 and ΔU = 0.55, *t*(25) = −1.71, *p* = 0.05.

As unpredicted effects are subject to alpha inflation in an ANOVA due to multiple testing^68^ all additional results in the analysis of Y-Axis and X-Axis should be interpreted with caution, and considered relative to a Bonferroni-adjusted alpha of 0.004. The analysis revealed an additional main effect of Trajectory that passed this threshold (*F*(1,79) = 197.5, *p* < 0.001, *η*_*p*_^2^ = 0.714), with perceived disappearance points of stimuli on arched trajectories being displaced further upwards (9.76 px, *t*(81) = 9.72, *p* < 0.001, *d* = 1.1) than for straight trajectories (0.67 px). This bias is consistent with the well-known predictive displacement in the direction of motion (e.g. further upwards for arched trajectories, but not for straight ones), known as the representational momentum effect. Interestingly, this forward displacement again differed between experimental groups, as indicated by an interaction of Trajectory and Experimental group that passed corrected thresholds (*F*(1,79) = 40.4, *p* < 0.001, *η*_*p*_^2^ = 0.506). Direct comparisons showed that the upwards displacements for arched trajectories were larger in Non-biological Ball (group 3) than the Biological Ball group (group 2; *F*(1,51) = 12.9, *p* = 0.001, *η*_*p*_^2^ = 0.203), which in turn were larger than in the Biological Hand group (group 1; *F*(1,54) = 31.9, *p* < 0.001, *η*_*p*_^2^ = 0.371). When analysing each experimental group separately, the upwards shift of straight trajectories was only present with ball stimuli, both when following biological, (*F*(1,26) = 100.5, *p* < 0.001, *η*_*p*_^2^ = 0.794), and non-biological trajectories, (*F*(1,79) = 147.7 *p* < 0.001, *η*_*p*_^2^ = 0.855), but not with moving hands, (*F*(1,28) = 2.35, *p* = 0.136, *η*_*p*_^2^ = 0.077). While not explicitly predicted, these displacements may reflect further changes to motion prediction depending on the presence of intentional cues. Balls, especially those that do not follow a biological motion trajectory, would be expected to continue on their upwards path, but hands would not when the goal of the reach is located towards the bottom, such as here. Nevertheless, due to the post-hoc nature of these findings, they should be treated with caution.

### X axis

**Figure 2:**
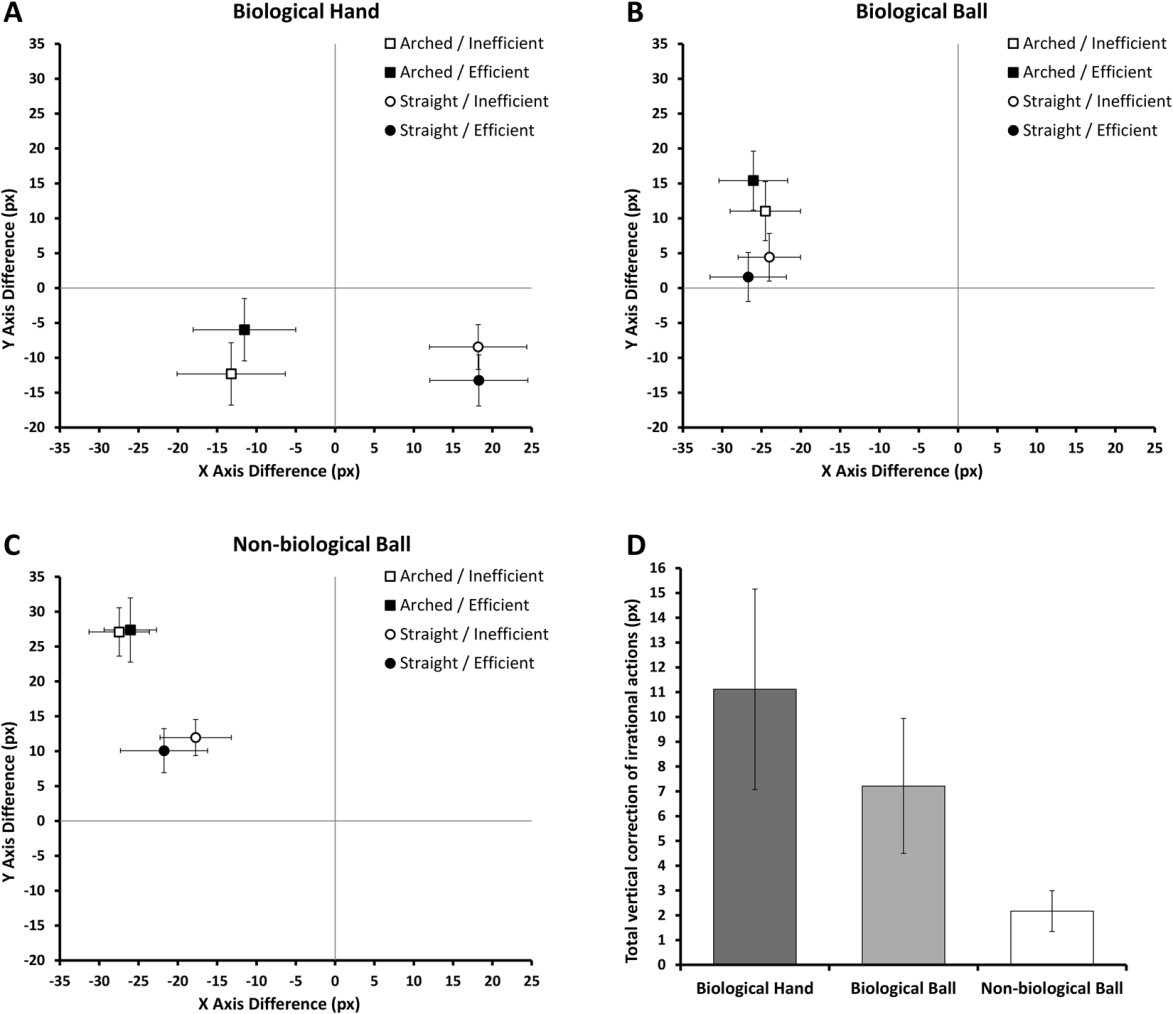
The Trajectory X Efficiency interactions for the Biological Hand (**A**), Biological Ball (**B**), and Non-biological Ball (**C**) groups. The difference between the real final position and the selected final position is plotted for the X axis and Y axis. The real final position on any given trial is at point 0,0, as indicated on each plot. Panel D depicts a comparison of the size of the Y axis interaction in pixels, equivalent to the total amount by which inefficient actions were corrected towards a more efficient trajectory. Error bars depict 95% confidence intervals.

We did not have any prediction for the X axis. All effects are therefore subject to alpha inflation in an ANOVA^68^ and should be interpreted with caution, relative to a Bonferroni-adjusted alpha of 0.004^68^. A main effect of Trajectory, (*F*(1,79) = 199.0, *p* < 0.001, *η*_*p*_^2^ = 0.716), passed this threshold, which was further qualified by an interaction of Trajectory and Experimental group (*F*(1,79) = 112.9, *p* < 0.001, *η*_p_^2^ = 0.741). As can be seen in Fig. 2, perceptual judgments of hands – but not balls – on arched trajectories were generally biased leftwards and rightwards for hands on straight trajectories. This difference replicates previous results^13^ and simply reflects stimulus differences between the hand shapes of the naturally recorded reaches on straight and arched trajectories, specifically the further rightwards centre of gravity for hands on straight trajectories, which biases location judgments^69^. No other effects passed the Bonferroni-adjusted thresholds of 0.004. Specifically, there was no main effect of Efficiency (*F*(1,79) = 0.4.66, *p* = 0.034, *η*_*p*_^2^ = 0.056), no interaction between Efficiency and Experimental group (*F*(1,79) = 5.42, *p* = 0.006, *η*_p_^2^ = 0.121), no interaction between Trajectory and Efficiency (*F*(1,79) = 5.15, *p* = 0.026, *η*_p_^2^ = 0.061) and no three-way interaction between Trajectory, Efficiency and Experimental group (*F*(1,79) = 1.25, *p* = 0.293, *η*_p_^2^ = 0.031).

### Testing for general differences in attention between groups

In an exploratory analysis, we tested whether the observed differences between groups can be explained by more general differences in attention towards the biological and non-biological stimuli. In particular, it is well-established that agentive stimuli with a biological motion profile attract attention^70–72^. To ensure that our results cannot be explained simply by more attentive perception of the more biological stimuli, we used the across-trial correlations between actual disappearance points and participants’ judgments that we used to identify participants that did not follow the task (i.e. if the reported x coordinates did not bear enough relationship to the actual coordinates). If participants attend more strongly to biological stimuli than to non-biological stimuli, one would expect their judgements to be more accurate and to more closely follow what was observed, resulting in smaller deviations for biological hand stimuli than for the other, less intentional stimulus types. We found no evidence for this prediction. While these correlations were generally high, they were, if anything, higher in the ball conditions in which participants’ judgments are less affected by their expectations (Hands, mean x *r* = 0.91, mean y *r* = 0.88; biological ball, mean x *r* = 0.95, mean y *r* = 0.93; non-biological ball, mean x *r* = 0.92, mean y *r* = 0.90). While this runs counter to the argument for decreased attention in the non-biological conditions, it is fully in line with our proposal of a stronger reliance on prior expectations as soon as intentions can be attributed to these stimuli. Indeed, as predicted from this hypothesis, participants’ across-trial correlations between actual and selected coordinates correlated negatively with how much they are affected by their expectations (*r* = −0.30, *p* = 0.006), even when gross between-groups differences are factored out via z standardization in each group (*r* = −0.31, *p* = 0.005). Thus, across all participants in the three groups, differences in the ability to track the actual disappearance points do no provide evidence for higher attention in the hand conditions but show, if anything, better accuracy in the non-biological groups, which can be explained by an (over-) reliance on prior expectations for stimuli that provide intentional cues.

## Discussion

Previous studies have shown that perceptual representations of observed actions are predictively biased towards the goals attributed to them^37,41,42,46–48^ and that these predictions are informed by the assumption of efficient action, reflecting the specific trajectories that would allow an actor to efficiently reach the inferred goal^13^. To investigate if these prior expectations emerge from assumptions about action intentionality, we asked participants to watch moving stimuli and to accurately report the object’s last seen position after it suddenly disappeared. We tested whether perceptual reports would again be predictively biased towards the expected trajectory^13,37,41,42^ but varied whether the stimulus was a hand with biological motion kinematics (i.e. bell-shaped velocity profile of reaching^60^), a non-agentive ball that travelled the same biological motion trajectory as the hand, or a ball travelling a non-biological trajectory.

Replicating our prior studies, perceptual reports of hand disappearance points were not veridical, but “corrected” towards the expected action kinematics of a rational, efficient actor. The perceived disappearance points of hands reaching straight towards an obstacle were reported higher than if the path was clear. Similarly, the perceived disappearance point of arched reaches was perceived lower if there was no obstacle to reach across, compared to when there was an obstacle. Importantly, our new data now show that these biases towards efficient action depend on cues to intentionality. The biases were numerically reduced when participants watched a non-intentional object – a ball – travel on the same biological motion trajectory, starting slowly and speeding up along, as if self-propelled. They were almost completely eliminated when the same ball was now seen travelling with a non-biological trajectory that nevertheless traversed, on average, the same path of motion as the hands, but did not show the characteristic bell-shaped velocity profile of goal-directed reaches^60^.

These results confirm first that, as in our prior studies, observers predict the ideal action trajectory a rational actor would take that is fully aware of all relevant environmental constraints. Second, they show that these predictions influenced the perceptual judgments of observed actions, subtly biasing them towards the most efficient trajectory. These findings are therefore in line with predictive processing models of social perception^12,15–17,26,41,42^, which assume that the perceptual experience of others’ actions emerges from an integration of bottom-up sensory information and prior assumptions about others’ goals and how they would (best) realise them. Our data now show, third, that when observing the behaviour of others these predictions of efficient action depend on bottom-up cues to intentionality derived from the objects’ semantics and its trajectory and motion profile. Both types of cues have been previously identified as a basis for attributing intentionality to observed agents in children^55–58^. The finding that these cues also modulate predictive biases towards efficient action in adult action observation directly supports the proposal that these predictions emerge from the attribution of intention to the observed actions^13,37,41,42^, which then inform their perceptual representation.

During everyday action observation these top-down influences can fulfil several important functions. First, they can disambiguate perception by compensating for the perceptual “blurring” during motion perception (i.e. motion sharpening^28,29^), or filling in missing steps of the input^30^. Second they can support planning of one’s own actions, allowing them to be coordinated with the others’ future behaviour or the end-state of their actions^73^. Finally, they can be compared to actual behaviour, triggering revisions of prior assumptions if prediction errors become too large^18,20^, signalling, for example, that a behaviour may not be intentional after all, or that the actor is not aware of all relevant environmental constraints (e.g., they may not have seen an obstacle). As such, they may underlie the proposed link between teleological perception of others’ behaviour and more sophisticated theory of mind and mentalizing processes^3^.

Further work now needs to resolve via which mechanisms cues of intentionality induce predictive biases towards efficient action. One possibility is that the biases emerge via predictive mechanisms in one’s own motor system^14–16,74,75^. On such views, people make higher-level “cognitive” attributions of intentions of others and then feed these goals into their own motor system to predict the kinematics they would need to achieve if they were in the actor’s place. Indeed, the perceptual effects observed here bear a striking similarity to similar motoric effects that can be measured when people watch others’ behaviour. Both behavioural and neuroimaging studies suggest that, during action observation, one’s own motor system does not only mirror the actually seen behaviour (e.g. a finger being depressed) but also the behaviour that is only *predicted* from the goals attributed to the actor, even if it is not actually observed^24,26^ (e.g., finger held up by a clamp^76^). Even if one watches an inanimate ball that one has experience of controlling oneself, one’s motor behaviour subtly captures both the ball’s actual trajectory and the trajectory one intended for it to travel on^77,78^. These motoric changes might therefore index the recruitment of such predictive (forward modelling) mechanisms that have evolved for the control of one’s own actions but are applied to the actions of others.

An alternative possibility is that attributions of intentionality are made within the (higher-level) perceptual system itself. It is well-known that the perceptual system itself can make sophisticated “unconscious inferences” about objects, extracting, for example, the real colour of a stimulus by subtracting out cues to shading and illumination^79^. In the same way, the perceptual system could use object and motion information (e.g., balls vs. hands; biological vs. non-biological motion profiles) to make inferences about the intentionality of a moving object^80^. Indeed, several imaging studies suggest that such cues to intentionality act on lower-level regions within higher-level visual cortex, such as the superior temporal sulcus^81,82^. Moreover, it well known that children can attribute intentionality to stimuli which are unlikely to engender motor activation, such as abstract geometric shapes or biomechanically impossible actions^83,84^ or that they process action efficiency before they have competence in the observed action^85,86^. Local interactions within the perceptual system could explain such observation. In such views, the motoric activation measured during action observation described above therefore does not reflect the origin of the perceptual effects, but a mere passive “motor resonance” that captures instead the changes to the action’s perceptual representation that has already occurred.

While we are sympathetic to both explanations^12^, and we do not deem them as mutually exclusive, our prior data seems to be more consistent with the latter, perceptual locus of effects. In our original study^42^, we observed that while attributing goals (e.g. to reach or withdraw) to others reliably biased perceptual measures towards these goals, the same was not true for when these action possibilities were motorically activated (i.e. by asking participants to make a forward or backwards movement with their own hand). While this conclusion is certainly preliminary, and needs to be supported by further studies, it makes a strong causal role of motoric processes unlikely.

Another question is how the present effects on perceptual judgments emerge. Several studies, both psychophysical and based on neuroimaging, have shown that predictions can exert downstream effects on early perceptual processes, across different modalities (e.g., vision^30,33^, audition^22^), providing sensory “templates” of expected stimulation^33^, or filling in missing information during apparent motion^30,34^. Others, however, argue that expectations influence primarily decision-related processes that integrate bottom-up with top-down information on all levels of the hierarchy^87,88^, or that they reflect attentional modulations of the response properties of neurons in early sensory areas^89,90^. Others argue that many of the psychophysical effects of expectation may in fact reflect testing artefacts or demand effects, when participants realise what is being tested^91,92^.

While the precise mechanism has to be confirmed, several aspects of prior studies^13^ imply a role in the action’s perceptual representation. First, when asked during piloting of the original set of studies, participants were unaware of the experimental hypotheses, arguing against demand effects. Second, the effects were present already very briefly (250 ms) after action offset, in psychophysical probe judgment tasks^13,37,41,42^ (for a review of similar findings in non-biological motion perception, see^40^) that has been shown to be relatively robust against cognitive control processes^93,94^. Third, and most importantly, the biases towards efficient action were disrupted by brief (560 ms.) dynamic visual noise masks that interfere with the re-entrant feedback from higher cortical areas with visual cortex that is required for the stabilisation of percepts for conscious access, during both perception^49,50,95,96^ and imagery^97^. The observed biases in perceptual judgments are therefore unlikely to stem from unspecific perceptual changes in memory or motor control^92^ (see^98^ for an example for perceptual changes in action memory). Instead, we propose that they either play a role in ongoing motion perception emerging from the re-current interactions between lower and higher visual regions involved in stabilising percepts and compensating for the substantial blurring during motion perception.

## Conclusions

The principle of efficient action allows observers to predict ideal reference trajectories that intentional actions will follow, given that the agent is fully aware of all relevant environmental constraints. The data presented here confirm that these predictions are at least partially perceptually represented and influence perceptual judgments of others actions, biasing them towards these expectations. They show that these predictions emerge from attributions of intentionality to the observed actor, triggered by the perception of biological “agentive” objects and kinematics that follow biological motion profiles.

## Data Availability

Data, code and materials are available at https://osf.io/x53uj/.
